# Intoxications aigues aux organophosphores chez la femme enceinte

**DOI:** 10.11604/pamj.2016.25.227.11041

**Published:** 2016-12-07

**Authors:** Mohamed Hafed Barhoumi, Badra Bannour, Tarek Barhoumi, Rami Jouini, Nadia Marwene, Mohamed Ridha Fatnassi

**Affiliations:** 1 Service d’Anesthésie et de Réanimation CHU Ibn El Jazzar Kairouan, Tunisie; 2 Service de Gynécologie et d’Obstétrique CHU Ibn El Jazzar Kairouan, Tunisie; 3 Direction Régionale de la Santé Kairouan, Tunisie

**Keywords:** Organophosphorés, intoxication aigue, grossesse, complications, fœtus, Organophosphorus, acute intoxication, pregnancy, complications, fetus

## Abstract

Les intoxications aiguës par les pesticides organophosphorés (OP) au cours de la grossesse sont des événements rares, peu décrites dans la littérature. A travers cette étude rétrospective, nous rapportons les résultats de sept cas d’ingestion suicidaire d’OP chez des femmes enceintes. Cette intoxication était le plus souvent grave. En effet, cinq des sept parturientes avaient un Glasgow initial < 9 et le POP score était ≥ 3 chez toutes les parturientes. Cinq patientes avaient nécessité une ventilation mécanique de durée moyenne de 3,4 jours. L’évolution était favorable pour toutes les patientes mais dans plus de la moitié des cas elle était défavorable pour le fœtus (mort fœtal in utéro). Deux mécanismes peuvent expliquer ces complications fœtales. Le premier est l’hypoxie fœtale associée ou non à un état de choc qui peuvent se traduire au niveau du RCF par une tachycardie ou des décélérations et aboutir ainsi à un décès in utero. Le deuxième mécanisme est le passage de ces pesticides à travers la barrière placentaire, ce qui pose un risque potentiel pour le fœtus par altération des systèmes enzymatiques des microsomes.

## Abstract (en)

Acute organophosphorus pesticides (OPs) poisoning during pregnancy are rare events, not well documented in the literature. We conducted a retrospective analysis of outcomes in seven cases of suicidal ingestion of OP in pregnant women. This intoxication was most often serious. Indeed, five of seven parturients had an initial Glasgow score < 9 and the POP score was ≥ 3 in all parturients. Five patients required mechanical ventilation for a mean duration of 3,4 days. All patients reported favorable outcomes but in more than half of the cases fetuses had unfavorable outcomes (fetal death in utero). Two mechanisms can explain these fetal complications. The first mechanism is fetal hypoxia, associated or not with a state of shock, which can be reflected in the Fetal Heart Rate (FHR) by tachycardia or decelerations and result in intrauterine fetal death. The second mechanism is the passage of these pesticides through the placental barrier representing a potential risk to the fetus due to the alteration of the microsomal enzyme systems.

## Introduction

Les organophosphorés (OP) sont des pesticides utilisés en milieu agricole comme insecticides. Ils sont des toxiques potentiellement létaux en cas d’intoxication aigue. Selon l’organisation mondiale de la santé, il y’a chaque année dans le monde trois millions d’empoisonnements graves par ces pesticides avec quelques 220000 décès [1]. Ce type d’intoxication se voit aussi bien chez l’homme que chez la femme et par conséquent il peut se produire chez la femme en âge de procréation, y compris celles qui sont enceintes. Toutefois, cette intoxication au cours de la grossesse est rarement rapportée dans la littérature. Le but de ce travail est de ressortir les particularités cliniques et évolutives des intoxications aigues aux OP chez la femme enceinte et d’essayer d’expliquer les mécanismes physiopathologiques des principales complications fœtales.

## Patient et observation

Il s’agit d’une étude rétrospective réalisée au service de réanimation polyvalente, durant une période de 3 ans, allant d’Avril 2013 à Avril 2015. Nous avons pu recruter sept dossiers cliniques de femmes enceintes, d’âges différents de grossesse, hospitalisées pour intoxication aigue aux OP. Le diagnostic a été basé, à l’admission, sur la présence d’un syndrome muscarinique et/ou nicotinique et/ou central, puis confirmé par le dosage de l’activité cholinestérasique plasmatique. Une échographie obstétricale a été réalisée pour toutes les parturientes avec évaluation de la vitalité et de l’état du fœtus. La prise en charge obstétricale a été dictée par la situation maternelle et fœtale. Le traitement a été fondé essentiellement sur la correction des détresses vitales et le traitement spécifique (Atropine et Pralidoxime) n’a pas été administré chez toutes les patientes. A partir du dossier médical, les paramètres sociodémographiques, cliniques et évolutifs des patientes et de leurs fœtus ont été recrutés. Le score POP (Peradeniya Organophosphorous Poisoning) (Tableau 1) [2] a été calculé pour évaluer la sévérité de l’intoxication. L’âge moyen de nos parturientes était de 23 ± 2 ans. Toutes les femmes étaient sans antécédents pathologiques notables. Toutes les intoxications étaient dans un but suicidaire. Le principal facteur déclenchant était le non désir de la grossesse. La majorité des patientes étaient primigestes (4/7; 57%). Le terme de la grossesse lors de l’ingestion des OP était en moyenne de 23,8 SA. La plupart des cas d’intoxication survenait au cours du 2^ème^ trimestre: 71,4% (5/7). L’association du syndrome muscarinique au syndrome nicotinique était le tableau clinique dominant. Le taux d’activité cholinestérasique plasmatique était effondré chez toutes les parturientes avec un score de POP toujours = 3. Cinq patientes ont nécessité une ventilation mécanique de durée moyenne de 3,4 jours. Une seule patiente a présenté un état de choc nécessitant sa mise sous catécholamine (Noradrénaline 3mg/H). L’évolution était favorable pour toutes les patientes mais dans plus de la moitié des cas elle était défavorable pour le fœtus (mort fœtal in utéro) (Tableau 2).

**Tableau 1:** Score de Peradeniya des intoxications aux organophosphorés

Paramètres	Critères	Score
	≥2	0
Taille des pupilles	<1	1
	Myosis serré	2
Fréquence respiratoire	<20/min	0
	≥20/min	1
	≥20/min avec cyanose	2
Fréquence cardiaque	>60/min	0
	41-60/min	1
	<40/min	2
fasciculation	Absente	0
	Presente généralisée/continue	1
	Présente généralisée et continue	2
Conscience	Conscient	0
	Réponse altérée a la demande	1
	Réponse absent à la demande	2
covulsion	absent	0
	present	1

0-3: intoxication minime

4-7: intoxication modéré

8-11: intoxication sévère

**Tableau 2:** Principales caractéristiques cliniques et évolutives des parturientes

Cas n°	Age (ans)	Parité	Age de la grossesse (SA)	POP Score	GCS	Ventilation mécanique, durée (jours)	Etat hémodynamique (FC, PA)	Activité cholinestérasique	Traitement spécifique, nature	Evolution de la grossesse
1	24	GIP0	21	3	15	Non	11/7, 100		Non	MAP/tocolyse/accouchement prématuré
2	25	GIIPI	A terme	8	7	Oui (2j)	18/10, 96		Oui (Pralidoxime)	Césarienne/mort périnatale
3	20	GIP0	14	5	7	Oui (1j)	11/7, 97		Oui (Atropine)	MFIU/aspiration
4	21	GIIPI	A terme	4	8	Oui (1j)	14/7, 102		Oui (Atropine-Pralidoxime)	Accouchement par voie basse
5	20	GIP0	20	3	9	Non	12/7, 120		Non	Accouchement à terme par voie basse
6	26	GIIAI	24	7	6	Oui (11j)	7/4, 104		Non	MFIU/avortement spontané
7	25	GIP0	18	4	8	Oui (2j)	13/9, 80		Non	MFIU/aspiration

*Semaines d’Aménorrhée ** Menace d’Accouchement Prématuré ***Mort Foetale In Utéro

## Discussion

L’empoisonnement intentionnel représente la méthode la plus fréquente de tentative de suicide non mortelle chez les femmes [3, 4]. Les produits utilisés pour l’intoxication varient selon les pays. En effet, l’ingestion d’une surdose de drogue ou de médicament a été signalée comme le principal mécanisme de tentatives de suicide chez les femmes enceintes en Californie (USA) [5]. Les insecticides organophosphorés dont l’accès est facile dans les pays en développement et notamment en Tunisie, sont largement utilisés dans les régions rurales. Ces produits dangereux entraînent des graves intoxications parfois mortelles [6]. Dans notre étude, la plupart des cas d’intoxication survenait au cours du 2^ème^ trimestre de la grossesse. Ceci concorde avec les données de la littérature qui rapportent une interaction significative entre l’âge de la grossesse et le taux d’intoxication. En effet, McClure et al ont avancés l’hypothèse d’une croissance de la conscience chez les femmes avec l’augmentation de l’âge gestationnel [7]. Ces irréversibles inhibiteurs de l’acétylcholinestérase prolongent et intensifient les effets de l’acétylcholine. L’accumulation de ce dernier, au niveau des récepteurs muscariniques, nicotiniques et au niveau des synapses du système nerveux, est responsable du tableau clinique des OP. Les signes cliniques peuvent se produire dans diverses combinaisons et peuvent se manifester à des moments différents selon le produit chimique, la dose et la voie d’exposition. Dans notre série, le tableau clinique dominant était représenté par l’association du syndrome muscarinique au syndrome nicotinique. La symptomatologie était le plus souvent grave. En effet, cinq des sept parturientes avaient un Glasgow initial < 9 et le POP score était toujours ≥ 3. Cette gravité peut être expliquée par la baisse physiologique de l’activité cholinestérasique plasmatique au cours de la grossesse. Howard et al ont montrés une baisse des cholinestérases chez les femmes en bonne santé au cours du premier trimestre de la grossesse. Ces cholinestérases reviennent à la normale au début du troisième trimestre [8].

L’intoxication aigue aux OP peut engager le pronostic vital aussi bien maternel que fœtal. L’exposition maternelle aigue ou chronique pré ou post conceptionnelle pourrait être à l’origine d’issues défavorables de grossesses (avortement, mort fœtal in utero, prématurité). Dans notre étude, l’évolution fœtale était défavorable dans plus de la moitié des cas. Sebe et al ont signalé un cas de mort fœtal à 19 SA chez une parturiente suite à une tentative de suicide aux OP [9]. Cependant, d’autre études ont constaté que la majorité des femmes qui ont survécu à la phase aigüe de l’intoxication, ont eu une grossesse de déroulement normal avec accouchement à terme [10, 11]. Deux mécanismes peuvent expliquer ces complications fœtales. L´hypoxie fœtale peut être due à la diminution de l´oxygène maternel et/ou à l’hypotension artérielle maternelle. Le fœtus est plus sensible que la mère à l´hypoxie et la gravité de son atteinte dépend de son stade de développement, de la durée et de la sévérité de l’hypoxie auquel il aura été exposé. Les tissus à haut métabolisme sont particulièrement sensibles à l´hypoxie tels que le cœur et le cerveau. Cette hypoxie peut se traduire au niveau du rythme cardiaque fœtal (RCF) par une tachycardie ou des décélérations et aboutir ainsi à un décès in utero [12]. Divers travaux suggèrent un effet de l’exposition maternelle aux pesticides sur le risque de mortalité intra-utérine et sur la diminution de la croissance fœtale. En effet, des études animales ont montré que les pesticides organophosphorés peuvent traverser la barrière placentaire, ce qui pose un risque potentiel pour le fœtus. Ce risque est lié à l’altération des systèmes enzymatiques des microsomes [11]. Le dosage de l’activité cholinestérasique plasmatique fœtale n’a cependant pas été fait chez aucune de nos patientes. La première étude constate que l’exposition préconceptionnelle à des herbicides de type chlorophénoxylé entraîne une augmentation modérée de fausses couches précoces alors que l’exposition à des herbicides tels que le glyphosate ou du type thiocarbamates est associée à des fausses couches tardives [13, 14]. L’autre montre une association significative entre prématurité et concentration sérique maternelle de dichlorodiphényldichloroéthylène (DDE) mesurée au cours de la grossesse [15].

L’intoxication aux composés OP pendant la grossesse est une urgence médicale qui nécessite souvent un traitement en unité de soins intensifs. Le traitement doit être effectué de manière appropriée avec la conscience des risques de tout médicament sur le fœtus. La littérature sur l’utilisation de l’atropine et de la Pralidoxime chez la femme enceinte est rare et ne fait pas partie de la stratégie thérapeutique générale [16, 17]. Dans une étude, l’effet de l’atropine à faible dose sur la fréquence cardiaque fœtale a été étudié. L’atropine agit sur le fonctionnement du système nerveux autonome du fœtus qui joue un rôle important dans l’adaptation du nouveau-né après l’accouchement [18]. Selon les études de cohorte de surveillance, l’atropine n’a pas montré aucun effet tératogène et son utilisation au cours de la grossesse ne devrait être retenue [16]. Le rôle des oximes dans l’amélioration de la survie et dans la réduction du recours à la ventilation mécanique après intoxication aux OP n’est pas évident [19]. Il n’existe aucune étude épidémiologique qui évalue le risque du Pralidoxime chez l’enceinte. Ces antidotes doivent être utilisés lorsqu’il existe un risque élevé de morbi-mortalité maternelle [16]. Dans notre série, le Pralidoxime n’a été utilisé que chez deux parturientes. L’administration a été faite en dehors de la période d’organogenèse et quand le bébé pourrait être accouché à tout moment pendant le traitement. Une étude réalisée aux États-Unis a remarqué une augmentation de certaines catégories de malformation congénitale, anomalies du système nerveux central et fentes labio-palatines, liées à l’exposition maternelle aux pesticides [20]. Aucun des fœtus dans notre étude n’avait une anomalie congénitale. Cependant, le suivi à long terme des nouveau-nés manquait dans notre population d’étude.

## Conclusion

Les tentatives d’autolyse par un pesticide organophosphoré sont rarement retrouvées chez la femme enceinte. Cette intoxication est grave et met en jeu le pronostic vital fœto-maternel. Comme dans la majorité des publications, notre population d’étude était trop petite pour tirer une conclusion définitive. Les efforts visant à réduire ces intoxications devrait inclure l’éducation du public sur l’utilisation des produits en vente libre et un meilleur soutien psychologique pour toute femme enceinte.
